# The Claims of Italy to the Invention of Lithotrity Vindicated

**Published:** 1841-01

**Authors:** 


### THE CLAIMS OF ITALY TO THE INVENTION OF LITHOTRITY VINDICATED. BY RAMBELLI.

Is lithotrity or lithotripsy a modern invention, belonging entirely to France? Let us examine history. In 1533 Alessandro Benedetti da Legnano described certain methods for the trituration of the stone in the bladder. In 1550 Alfonsi Ferri, a Neapolitan, invented an instrument, which he called after himself Alfonsino, for the extraction of bullets from gun-shot wounds, and which gave the idea of that which was in the sequel invented to extract and break the stone. In 1614 Baronio da Cremona first adopted the injection into the bladder of liquids to dissolve calculi, such as lemon-juice, &c., and thus far preceded Hales, Langrish, Bater, Rutherford, Fourcroy, Vauquelin, and others. In 1626 Santorio invented a canula, which contained within it an instrument with three branches, which opened for the extraction of foreign bodies in the bladder or the urethra; and within this instrument he passed a stiletto for the purpose of perforating the calculus when he could not extract it entire. In 1679 Anton Filippo Ciucci, a surgeon of Arezzo, published his *Promptuarium chirurgicum*, from which it is quite evident that he preceded all others in the invention of lithotripsy.

Ciucci flourished in 1670, and practised surgery in Macerato with reputation, after having passed two years of practice at Rome under Dr. Giovanni Trullo. Besides the Promptuarium, he wrote a treatise on the circulation of the blood, with an appendix on the plague, and dedicated it to Francisco Redi. In the second part of his Promptuarium, he asserts that among the many remedies invented for urinary calculi, there is not any to compare with the *tenacula tricuspis*; which instrument, he says, is not altogether different "a pede gryphio cum quo molæ ab utero educuntur," but is unlike it, "quia istud non habet acumina mucronata, sed retusa." He subjoins that "debet chirurgus et tenacula frustula diligenter quærere;" and that for the remaining heat and pain "possumus injicere modicum lactis .... et sic absque incisione ad pristinam sanitatem languentem conducemus .... cito, tuto, et jucunde." Ciucci had such confidence in his method, that he himself submitted to it three times. He gives a representation of his tenacula, which, as already said, is very similar to the ball-extractor of Ferri.

Lastly, Santarelli, a surgeon of Rome, in his researches to facilitate catheterism (Vienna, 1795), proposes the straight catheter. Professor Cittadini, of Arezzo, compares the instrument of Ciucci, invented in 1679, with that of Civiale in 1823; he recognizes a perfect similarity between the two, except that that of Ciucci is solid and full, and that of Civiale is hollowed for the passage of the lithotritor, which acts by boring and destroying the calculus.

From these circumstances it may be concluded that this most important invention does not belong either to Civiale or Gruithuisen (1813), or Egerton (1819), or Leroy d'Etiolles (1821), or Amussat (1822). All of these dispute the honour of having preceded Civiale in the invention and description of the operation of lithotrity; an operation which, it is evident, was imagined and executed by two illustrious Italians, Santorio and Ciucci.

*Il Filiatre-Sebezio. Marzo*, 1840. (From the *Annali Medico-Chirurgici.*)

---

